# Guidelines (1988) for listing specifications of centrifuges

**DOI:** 10.1155/S1463924689000064

**Published:** 1989

**Authors:** A. Uldall, P. Trier Damgaard, E. Magnussen, O. Drachmann, J. C. Rigg

**Affiliations:** Department of Clinical Chemistry University of Copenhagen Herlev Hospital Herlev Ringvej 75, Herlev DK 2730 Denmark


Journal of Automatic Chemistry Vol. 11, No. 1 (January–February 1989), pp. 28–31

### Guidelines (1988) for listing specifications of centrifuges

### International Federation of Clinical Chemistry Scientific Committee Expert Panel on Analytical Systems[1]
### IFCC Document, Stage 2, Draft 1, December 1987

**Prepared for publication by A. Uldall (DK), P. Trier Damgaard (DK), E. Magnussen (DK), O. Drachmann (DK) and J. C. Rigg (NL)**

Comments on the Guidelines and reprint request should be sent to:

*Adam Uldall, Dr. Pharm., Ph.D., Department of Clinical Chemistry, University of Copenhagen, Herlev Hospital, Herlev Ringvej 75, DK 2730 Herlev, Denmark*

### (1) Introduction

(1.1) Centrifuges available for use in the clinical chemistry laboratory vary considerably in their complexity and safety. The Expert Panel considered that guidelines for the preparation of specifications in a standard format should be available to assist with the understanding of use of each instrument.

The guidelines are based on British Standard 4402:

*Specification for Safety requirements for laboratory centrifuges 1982* [1] and on Copenhagen County Hospital Service: *Recommendation Regarding Laboratory Centrifuges 1979* [2].

The nomenclature used in the guidelines is defined in Appendix A. Quantities and units with regard to the use of centrifuges will be described in a later paper (section 1.5).

(1.2) Manufacturers using the guidelines to describe the specifications of centrifuges should state that they have done so, but if such a statement is made, information on all items listed should be provided unless inapplicable to the instrument being described.

(1.3) Manufacturers should provide the information in the order listed here in the interests of uniformity.

(1.4) Similar guidelines have been produced for spectrometers [3], chemical analysers [4], flame emission spectrometers [5], atomic absorption spectrometers [6], and are in preparation for nephelometers.

(1.5) The present guidelines on laboratory centrifuges are the first of a series dealing with centrifuge safety, including selection of centrifuges, safety instructions for use, and preventative maintenance.

[1] *Expert Panel Members: C. A. Burtis, Chairman (US); P. A. Akinyanyo (NI), T. Sasaki (JP).*

### (2) General information

(2.1) Date of completion of the listing by the manufacturer.

(2.2) Name and address of manufacturer and, if applicable, of national agency.

(2.3) Name of the centrifuge and model designation.

(2.4) Brief history, if considered to be of interest to potential customers.

### (3) Summary of general specifications

(3.1) Special functions and features (maximum 100 words).

(3.2) External dimensions: length, breadth and height.

(3.3) Mass of the centrifuge.

(3.4) Diameter, depth and volume of the centrifuge chamber.

(3.5) Electrical specifications: potential difference (V), electrical current (A) and frequency of alternating current (Hz). State minima and maxima for optimum operation.

(3.6) Power consumption of motor.

(3.7) Whether centrifuge chamber can be heated or cooled.

(3.8) For thermostated centrifuge chamber, state adjustable temperature range, and the allowed variation of temperature within one setting as well as its accuracy.

(3.9) Evacuated centrifuge chamber and display of air pressure.

(3.10) Selector for rotational frequency.

(3.11) Timer, programmer, and indicator which shows when the lid may be opened.

(3.12) Indicator of actual revolution frequency.

(3.13) Indicator of centrifugal acceleration or massic centrifugal force (definitions to be published in list of centrifugal quantities).

(3.14) Indicator of temperature of centrifuge chamber.

(3.15) Mechanical safety devices.

(3.15.1) Automatic shutdown device for the motor when the access cover is opened.

0142–0453/89 $3.00 © 1989 Taylor & Francis Ltd.

(3.15.2) Automatic cover lock.

(3.15.3) Override release system for maintenance.

(3.15.4) Out-of-balance switch.

(3.15.5) Single or double speed-limiting devices or equivalent construction; any speed-limiting device that responds individually and adequately on each type of rotor assembly.

(3.15.6) Documentation of the mechanical safety of the guard barrier, base, top, access, cover with hinges and lock, cabinet and fixings. Name any official standard the centrifuge complies with, (for example [1]).

(3.15.7) Sealed centrifuge chamber to prevent fragments from escaping during a breakdown of the rotation assembly.

(3.15.8) Centrifuge mountings to bench or floor.

(3.16) Safety features for flammable substances or for biological hazardous materials (rotor assembly is referred to section 3.17.9).

(3.16.1) Ignition protected motor and electronics.

(3.16.2) Ventilation of the centrifuge chamber, air filters.

(3.16.3) Decontamination of centrifuge chamber.

(3.17) Rotor assemblies

For each type the following information should be provided:

(3.17.1) Name and intended use.

(3.17.2) Mass and diameter of the complete rotor assembly (buckets in horizontal position).

(3.17.3) Number of vials, bags or tubes; the size should be specified. Total volume of material (volumic mass density $\leq 1{\cdot}2$ kg/l), which may be centrifuged in one run.

(3.17.4) Maximum permissible rotational frequency; the corresponding centrifugal acceleration in top and bottom of the vials; bags or tubes; and the maximal kinetic energy of the fully loaded rotor assembly.

(3.17.5) Time from start until maximal rotational frequency is achieved.

(3.17.6) Braking time from maximal rotational frequency.

(3.17.7) For an angular rotor, the angle between the vials and the rotation axis.

(3.17.8) Wind-shielded rotor assembly.

(3.17.9) Sealed rotation assembly and/or buckets.

(3.17.10) Documentation of the mechanical safety of rotor head, buckets and the mounting system for the buckets, for instance by referring to any official standard the evaluation complies with.

(3.17.11) Resistance to acid, alkali, cleaning agents and decontamination by heat.

(3.17.12) Accessories such as trays or racks from other manufacturers that can be used in the rotor assembly.

(3.17.13) K-factor for fixed angle rotors to ultracentrifuges [8].

### (4) Other options

### (5) Documentation available to the user

(5.1) Operating manual.

(5.2) Maintenance manual including list of spare parts with their catalogue numbers.

(5.3) Full service manual.

(5.4) List of available evaluations.

### (6) Information to be supplied locally and perhaps separately

(6.1) Costs.

(6.1.1) Purchase price.

(6.1.2) Average running power consumption.

(6.1.3) Maintenance cost, for instance of annual contract or hourly rates.

(6.2) Maintenance, service and parts available and training [7].

(6.3) List of spare parts and consumables held locally by manufacturer or agent.

(6.4) List of recommended parts and consumables to be held by the user.

(6.5) Conditions of guarantee

### References

1. British Standards Institution, *Specification for Safety Requirements for Laboratory Centrifuges* 4 pages (BS 4402, 1982).
2. Copenhagen County Hospital Service. Centrifuge committee. *Recommendation Regarding Laboratory Centrifuges 1979* (Københavns Amtskommune, Sygehusdirektoratet, København, 1979).
3. Haeckel, R., Collombel, Ch., Geary, T. D., Mitchell, F. L., Nadeau, R. G. and Okuda, K. Provisional guidelines (1979) for listing specifications of spectrometers in clinical chemistry. *Clinica Chimica Acta,* **103**; 249F–258F; *Journal of Clinical Chemistry and Clinical Biochemistry*, **18** (1980), 445–449; *Clinical Chemistry*, **27** (1981), 187–191.
4. Okuda, K., Collombel, Ch., Geary, T. D., Haeckel, R., Mitchell, F. L. and Nadeau, R. G. Provisional guidelines (1980) for listing specifications of clinical chemical analysers. *Journal of Clinical Chemistry and Clinical Biochemistry*, **18** (1980), 947–951; *Clinica Chimica Acta,* **119** (1982), 353F–362F.
5. Bechtler, G., Epstein, M. S., Geary, T. D., Havemann, W. and Attoe, P. Provisional guidelines (1981) for listing specifications of flame emission spectrometers. *Journal of Clinical Chemistry and Clinical Biochemistry*, **20** (1982), 259–261.
6. Epstein, M. S., Geary, T. D., Gower, G., Tausch, W., Mills, K. J. and Polt, D. Provisional guidelines (1981) for listing specifications of atomic absorption spectrometers. *Journal of Clinical Chemistry and Clinical Biochemistry*, **20** (1982), 263–266.
7. National Committee for Clinical Laboratory Standards AS1-I, *Preparation of Manuals for Installation, Operation and Repair of Laboratory Instruments* (NCCLS, 771 East Lancaster Avenue, Villanova, Pennsylvania 19088, USA).

8. Richwood, D. (Ed), *Centrifugation: a Practical Approach* (IRL Press Limited, Oxford and Washington, D.C., 1984, 2nd ed.) I–XII, pp. 1–352.

### APPENDIX A

### Nomenclature of centrifuges

The parts of centrifuges are named and described according to the alphabetic list below. Most parts are also illustrated in figures 1 and 2. In restricted contexts, the word 'centrifuge' may be omitted as qualification to certain terms.

*Angle head (of centrifuge):* a centrifuge head into which tubes or tube holders can be placed at an angle that is maintained during rotation.

*Brake:* mechanical or electrical device to reduce rotational frequency.

*Bucket (of a centrifuge):* specimen holder or a carrier for specimen holder mounted directly to swing-out head.

*Centrifuge:* a motor-driven machine used in chemistry to separate components, or to alter the local distribution of components of a system by means of centrifugal acceleration in rapidly rotating vessels.

*(Centrifuge) adaptor:* a fitment allowing specimen holders of different sizes to be placed in the rotor assembly.

*(Centrifuge) casing:* the cabinet of the centrifuge, including top access cover and bottom. Usually the casing includes a guard barrier or may surround a guard barrier.

*(Centrifuge) chamber:* the space enclosed by the casing of a centrifuge in which the rotor assembly rotates.

*(Centrifuge) head* (rotor centre piece): the part of the centrifuge that is mounted directly to the rotating axis and rotates. Special types are the angle head (see figure 1) and the swing-out head (see figure 2). For the latter type, the buckets are not part of the head.

*Cover lock:* safety lock or snap lock. In centrifuges of maximal kinetic energy usually less than 1 kJ, the snap lock switches off the power when it is opened.

*Guard barrier:* a mechanically reinforced part of the casing or a separate strong shield surrounding the centrifuge chamber.

*Insert* (to specimen-holder assembly): special adaptors for different types of tubes, thus a certain specimen-holder assembly can be used for different tubes.

*Indicator* (on equipment): a device indicating the value of an operating variable for a piece of equipment, for instance rotational frequency of a centrifuge.

*Out-of-balance switch:* a switch to cut out the motor of a centrifuge that is out of balance.

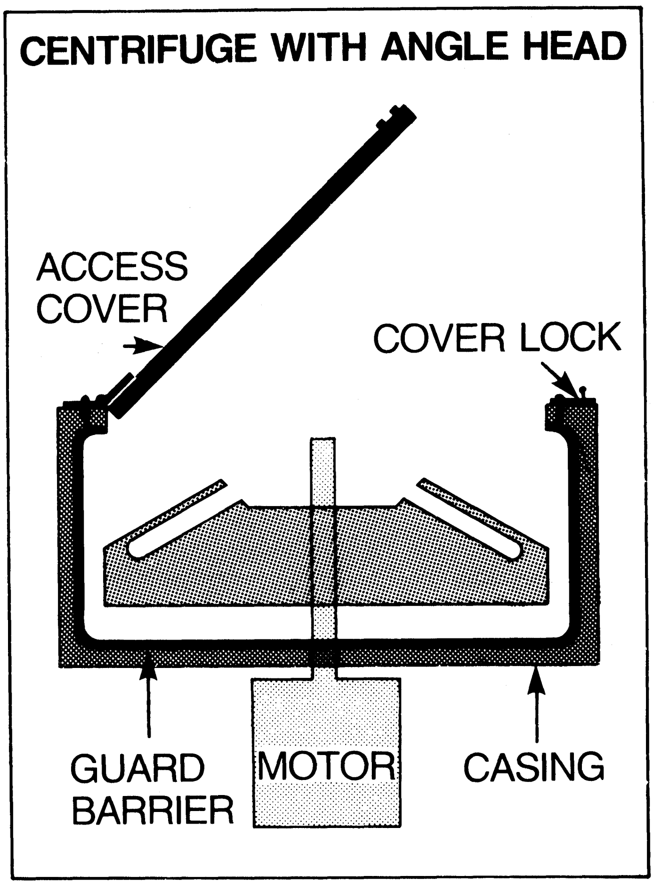

*Figure 1. Centrifuge with angle head.*

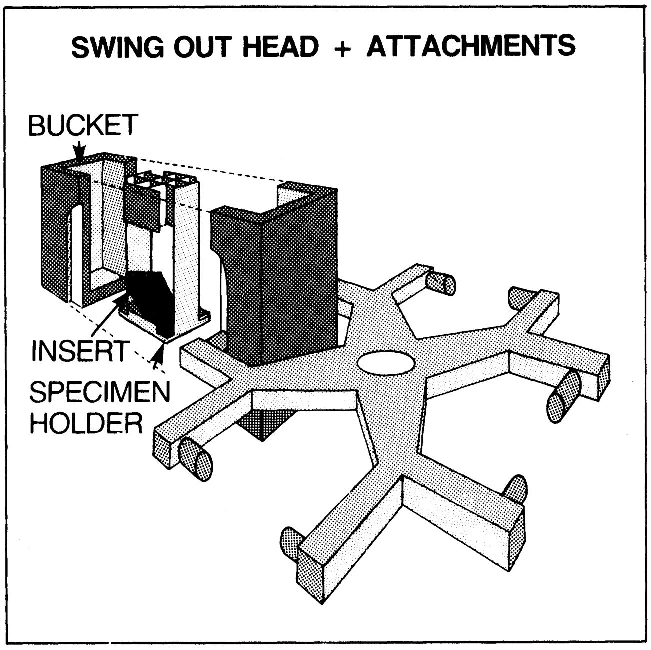

*Figure 2. Swing out head and attachments.*

*Rotor assembly (of centrifuge):* the centrifuge head with any tube holders, specimen holders, adaptors, inserts and lid for sealed head or buckets.

*Safety lock:* a device, usually electrically operated, preventing opening of a centrifuge during operation. Safety locks are used mainly in centrifuges of maximum kinetic energy greater than 1 kJ.

*Sealed rotor assembly (or bucket):* special rotor assembly or bucket of some centrifuges to reduce the hazard of flammable or biologically dangerous fluids being forced out, for instance as an aerosol.

*Selector* (of rotational frequency): an operating device for adjustment of an operational variable, in the present context rotational frequency, before switching on a piece of equipment.

*Specimen holder:* container or support for a specimen in scientific or technical investigation, for instance a test-tube, a flask, a centrifuge tube or bucket, or a microscopic slide.

*Specimen-holder assembly:* the complete assembly in which the specimens are housed.

*Swing-out head (of centrifuge):* a centrifuge head in which the specimen holders change their angle in relation to the axis of spin during rotation.

---

### THIRD INTERNATIONAL SYMPOSIUM ON KINETICS IN ANALYTICAL CHEMISTRY

*To be held in Dubrovnik, Yugoslavia, from 25 to 28 September 1989*

The International Symposium on Kinetics in Analytical Chemistry 1989 ( KAC'89 ) will be held in Cavtat (Dubrovnik) from 25 September to 28 September 1989. The Symposium is the next in the series of triennial conferences, initiated in Cordoba in Spain in 1983, as a result of rapid development in kinetic methods of analysis. The success of the first conference prompted the second meeting which was held in Preveza, Greece in 1986.

*Scientific programme*
The scientific programme will be organized around plenary, invited and contributed papers and posters. The scope of the symposium will be similar to that of the earlier ones, and will include catalytic (enzymatic or non-enzymatic) and non-catalytic methods, differential reaction rate methods, unsegmented flow methods, and any other kinetic aspect of analytical interest.

*The Scientific Committee includes:*
Professor H. A. Mottola (Stillwater, Oklahoma, USA)
Professor G. Werner (Leipzig, GDR)
Professor M. Valcárcel (Córdoba, Spain)
Professor M. I. Karayannis (loannina, Greece)
Professor G. A. Milovanović (Belgrade, Yugoslavia)
Professor F. F. Gaál (Novi Sad, Yugoslavia)

*Language*
The offical language of the Symposium will be English.

*General information and social programme*
The Symposium will be held in the Croatia Hotel in Cavtat, a small town on the Adriatic, close to Dubrovnik. A varied social programme is being prepared to accompany KAC'89 and will include a Welcome Party, a Symposium Banquet and several tours. Post-Symposium excursions will be also arranged.

*Travel Agency*
The official travel agency for KAC'89 (accomodation and post-symposium tours) will be YUGOTOURS, Congress Department Dure Dakovića 31, 11 000 Belgrade, Yugoslavia.

*Further information from Professor Gordana A. Milovanović, Department of Chemistry, University of Belgrade, KAC, P. O. Box 550, 11001 Belgrade, Yugoslavia.*

